# Stroboscopic vision and sustained attention during coincidence-anticipation

**DOI:** 10.1038/s41598-017-18092-5

**Published:** 2017-12-20

**Authors:** Rafael Ballester, Florentino Huertas, Makoto Uji, Simon J. Bennett

**Affiliations:** 1 Department of Athletic Training, Universidad Católica de Valencia “San Vicente Mártir”, Valencia, Spain; 2 Research Institute for Sport & Exercise Sciences, Liverpool John Moores University, Liverpool, UK

## Abstract

We compared coincidence-anticipation performance in normal vision and stroboscopic vision as a function of time-on-task. Participants estimated the arrival time of a real object that moved with constant acceleration (−0.7, 0, +0.7 m/s^2^) in a pseudo-randomised order across 4 blocks of 30 trials in both vision conditions, received in a counter-balanced order. Participants (n = 20) became more errorful (accuracy and variability) in the normal vision condition as a function of time-on-task, whereas performance was maintained in the stroboscopic vision condition. We interpret these data as showing that participants failed to maintain coincidence-anticipation performance in the normal vision condition due to monotony and attentional underload. In contrast, the stroboscopic vision condition placed a greater demand on visual-spatial memory for motion extrapolation, and thus participants did not experience the typical vigilance decrement in performance. While short-term adaptation effects from practicing in stroboscopic vision are promising, future work needs to consider for how long participants can maintain effortful processing, and whether there are negative carry-over effects from cognitive fatigue when transferring to normal vision.

## Introduction

The human visual system typically receives an intermittent flow of incoming information due to blinks, saccades and periods of transient occlusion when an object-of-interest disappears from view behind another object or surface (e.g., as the ball is obscured by the defensive players during a free kick in soccer). This usually goes unnoticed, with the intermittent input transformed into a unified and continuous perceptual experience. However, even when there are longer periods of occlusion (e.g., artificial manipulation using stroboscopic vision eyewear), relevant information can be gained from intermittent visual samples to provide sufficient information for successful performance of precision interceptive actions^1^. Recently, it has been reported that practicing in such vision conditions can facilitate sports-specific skills in ice-hockey^2^ and baseball^3^. Analogous to altitude training for the endurance athlete^4^, the premise is that practicing in stroboscopic vision encourages visual-cognitive processes to adapt in order to cope with the suboptimal information available. Processes shown to transfer positively when vision is subsequently restored to normal include short-term visual memory^5^, coincidence-anticipation timing^6^, and motion coherence and attention in central vision^7^.

Continuing with the analogy of altitude training, it follows that practicing in stroboscopic vision is effortful and attentionally demanding. Indeed, anecdotal reports suggest that participants exhibit more focussed attention on an approaching object when practicing catching tasks in stroboscopic vision^8^. This is consistent with related empirical work that has shown an overall increase in attention (i.e., “high-beams” effect) in order to maintain a persistent visual-spatial memory of relevant stimulus locations (i.e., object and distractors) when vision is intermittently occluded^9^. It is important to recognize, however, that a high attentional load and effortful processing cannot be maintained indefinitely. In accord with the overload hypothesis^10^, it follows that a high attentional load can eventually lead to the depletion of attentional resources and a decrement in performance. This has implications for the design of stroboscopic vision training programmes, which to date have used both experimenter-determined (e.g., 25 minutes^5^, 5–7 minutes^6^, and self-determined (e.g., 10–45 minutes^2^) exposure duration.

In the current study we sought to determine the effect of stroboscopic vision on attentional allocation while performing coincidence-anticipation timing, which is a key element to many daily life activities such as driving or in different sporting disciplines where it is necessary to avoid or intercept moving objects^11^. Rather than using a probe-reaction procedure to determine the amount of attention used when performing coincidence-anticipation timing in stroboscopic vision compared to normal vision, we were interested to know if stroboscopic vision influences the ability to sustain attention as a function of time-on-task. Therefore, we adopted the method used for testing psychomotor vigilance, whereby participants are required to sustain attention over time in order to respond efficiently to repeated presentation of the imperative stimulus^12^. Specifically, we compared vigilance in a normal vision condition and a stroboscopic vision condition (4 Hz) while performing repeated trials of a coincidence-anticipation task in which the object moved with constant acceleration (−0.7, 0, +0.7 m/s^2^). We hypothesised that participants would exhibit deterioration in performance (accuracy and variability) as a function of time-on-task in the normal vision condition due to monotony and attentional underload^13^. Conversely, we hypothesised that the greater demand on visual-spatial memory for motion extrapolation in the stroboscopic vision condition would enable participants to sustain attention and thus offset the typical vigilance decrement.

## Methods

### Participants

Twenty male undergraduate students (*M* = 23.15 years of age, *SD* = 2.35) volunteered to take part in the study. All participants reported having normal or corrected-to-normal vision. Participants were provided with general information about the task and stimulus prior to giving informed written consent. All procedures were conducted in accordance with the Declaration of Helsinki and were approved by the Liverpool John Moores University Research Ethics Committee.

### Apparatus, Task and Procedure

#### Coincidence-Anticipation

Participants were required to press a button mounted in a hand-held joystick at the moment an object (single red LED of 5 mm diameter) that moved along a 3 m linear track (HEPCO) reached a fixed target position. The target comprised two red LEDs (5 mm diameter) mounted on either side of the track. The object was attached to a sled that was moved along the linear track by a stepper motor controlled by in-house routines implemented in MATLAB (The Mathworks, Inc., MA, USA). The object moved with constant acceleration (−0.7, 0, +0.7 m/s^2^) such that it reached the target after 1000 ms moving with a velocity of 1.25 m/s. It then continued to move with the same acceleration for a further 100 ms, after which it was brought to a standstill. The target remained stationary for 2000 ms, and then moved slowly back to the start position for the next trial. The moment the button was pressed and the sled reached a switch located coincident with the target were recorded via a data acquisition card (NI PCI-6035E) and stored for offline analysis.

Participants performed the coincidence-anticipation task in a normal vision condition and stroboscopic vision condition. In the latter, participants wore eyewear (Nike Vapor Strobe®) with LCD lenses that cycled between “open” and “closed” states. The “open” state had a fixed duration of 100 ms, whereas the “closed” state could be set at one of eight levels. Following previous work on stroboscopic vision during coincidence-anticipation^6^, here we selected level 3 for the closed state, which had a duration of 150 ms (i.e., 4 Hz cycling rate). In the “closed” state the lenses are less transparent and thereby are likely to perturb perception of motion and form (see discussion). Effectively, in the “closed” state the lenses act as neutral density filters and thus reduce light transmission. Under ambient room lighting (625 lux), which was used throughout experimental testing, a digital light meter (Lutron LX-1108, Taipei, Taiwan) located directly behind the lens of the stroboscopic vision eyewear indicated the illuminance was 128 lux in the “closed” state. An illuminance of 100 lux is similar to that of a “very dark overcast day”^14^, while 320 lux is the minimum illuminance for office lighting recommended by the US Department of Labour. We were unable to reliably measure illuminance when the stroboscopic eyewear were in the “open” state (100 ms), although the lenses were sufficiently transparent that participants reported having normal visibility. To ensure that participants in the normal vision condition believed they were the subject of an intervention, and thus experienced similar expectation effects as the stroboscopic vision condition (i.e., Hawthorne and/or placebo effect), they performed the coincidence-anticipation task while wearing a pair of NVIDIA LCD shutter glasses (Expressway Santa Clara, CA, USA). These were not switched on and connected to a 3D graphics card, and thus permitted light transmission of 239 lux. While illuminance was reduced compared to that of ambient lighting, participants reported having an uninterrupted view of the moving object during the coincidence-anticipation task.

Prior to the commencing experimental testing, the experimenter explained the procedure and provided the participant with the necessary eyewear. Half of the participants performed the coincidence-anticipation task in the stroboscopic vision condition followed by the normal vision condition, whereas the other half performed the normal vision condition followed by the stroboscopic vision condition. The participant next performed 10 familiarization trials, followed by 4 blocks of 30 experimental trials. Within each block, the level of acceleration was pseudo-randomly ordered to encourage participants to use the available visual information (e.g., not respond at a fixed distance) and to minimize boredom associated with repeated attempts with the same motion. To prevent a learning effect with respect to acceleration that could have influenced allocation of attention, knowledge of results on coincidence-anticipation accuracy was not communicated to the participant. The duration to complete each vision condition was between ten and eleven minutes depending on the participant’s response time to each trial, and was thus similar to previous studies that have shown a vigilance decrement when completing a computer-based reaction time (RT) task (see below) for an extended number of trials without a break.

#### Psychomotor Vigilance

**Figure 1:**
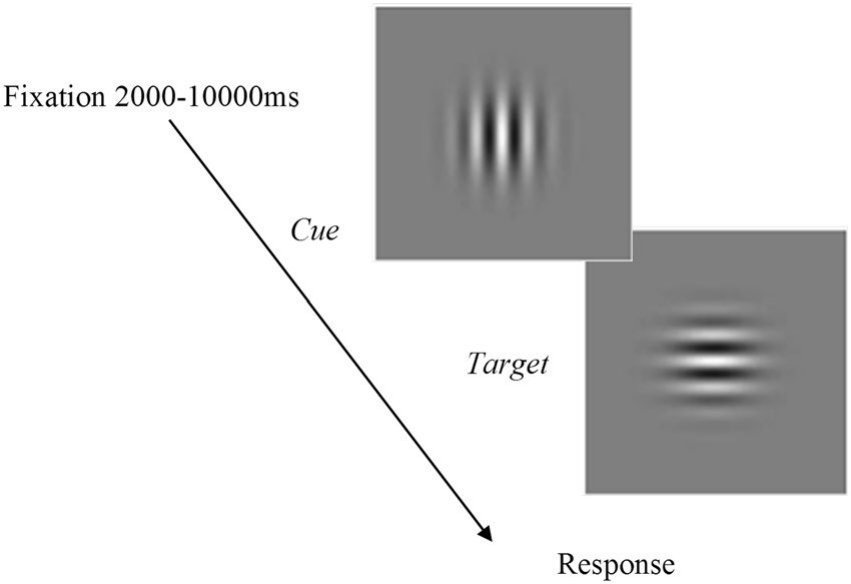
Temporal course of the stimuli presentation in the PVT.

Between completing the coincidence-anticipation task in the normal vision and stroboscopic vision conditions, participants performed a computer-based psychomotor vigilance task (PVT). The presentation of stimuli, timing operation, and collection of responses was controlled by E-Prime software (Psychology Software Tools, Pittsburgh, PA, USA) running on a desktop computer (Dell OptiPlex). The PVT required participants to respond, as rapidly as possible, to a visual stimulus that appeared on a computer monitor located 50 cm from where they were seated. During each trial of the PVT, a Gabor patch (4.20° × 4.20°) was presented with a horizontal orientation against a grey background at the center of the screen. Then, after a random time interval between 2000 ms and 10000 ms, the orientation of the Gabor patch was abruptly switched to vertical (see Fig. 1). Participants were instructed to respond to this change of orientation as quickly as possible by pressing the space bar on a keyboard (Razr Lycosa, 1000 Hz polling) with the index finger of their dominant hand. Feedback of the response time was displayed on the screen after each trial during a 300 ms inter-trial interval. If no response was given within 5000 ms of changing the orientation of the Gabor patch, the message “You did not answer” appeared on the screen and the next trial began. The PVT lasted for 9 minutes without interruptions and is accepted to provide a simple and reliable measure of vigilance given the monotonous, repetitive, and unpredictable nature of the target onset^15^. It has been reported that failures (e.g., slowing of RT or increase in lapses) in vigilance performance in the PVT can occur within 5 minutes in adults^15,16^, and even shorter duration in adolescents and children^17–19^ However, we decided to follow the original developers’ recommendation of a 9 minute PVT^20^, which also approximated the duration of coincidence-anticipation task and thus permitted between-task comparison.

### Data Analysis

#### Coincidence-Anticipation

We first calculated the signed error on each trial between button press and object arrival at the target. Response with an absolute value of >300 ms were classified as outliers and removed (<1.0%) from additional analyses^11^. From the remaining trials, we calculated intra-participant mean constant error (accuracy) and variable error (variability) for each level of object acceleration in each of the four blocks. The intra-participant mean data were submitted to separate 2 Vision (normal, stroboscopic) x 3 Acceleration (−0.7, 0, + 0.7 m/s^2^) by 4 Block (1, 2, 3, 4) repeated measures ANOVA. In cases where Mauchley’s sphericity test was significant, Greenhouse-Geisser corrections were applied. Tukey’s Honestly Significant Difference (HSD) tests were then used to determine the origin of any significant main and interaction effects.

#### Psychomotor Vigilance

Intra-participant mean (accuracy) and standard deviation (variability) of RT was calculated for consecutive 3 minute intervals of the 9 minute total task duration. Trials with RT below 100 ms (<1.0%) were considered to be anticipation errors and therefore discarded from the analysis^15^. The intra-participant mean and standard deviation data of RT submitted to a one-way ANOVA with Block (1, 2, 3) as a repeated measure. Tukey HSD post-hoc tests were used to investigate the significant main effect.

## Results

### Coincidence-Anticipation

**Figure 2:**
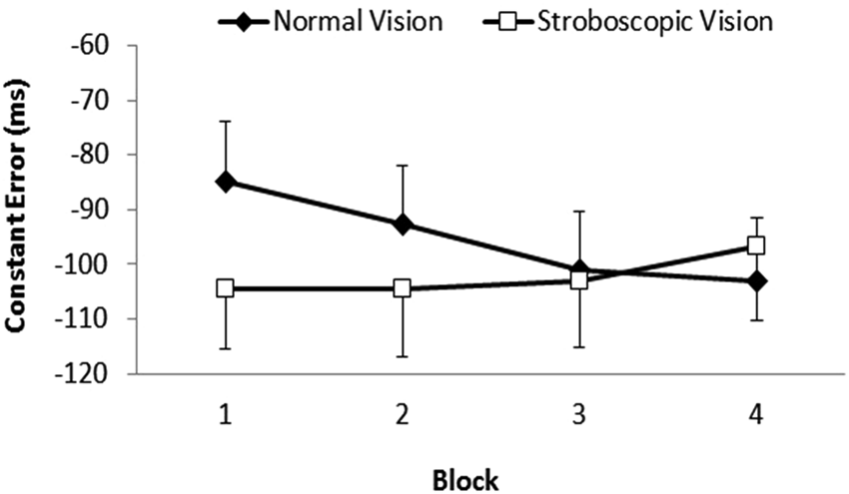
Group mean constant error as a function of time-on-task (Block 1–4) for the normal vision and stroboscopic vision conditions. Vertical bars represent standard errors of the mean.

For constant error there was a significant main effect of Acceleration, *F*(1.26,24.03) = 56.62, *p* < 0.001, η^2^_partial_ = 0.75. Participants exhibited greater underestimation of object arrival when it decelerated (−117 ms) compared to when it had constant velocity (−101 ms) or accelerated (−78 ms), *p* < 0.001. There was no main effect of Block, *F*(3,57) = 2.47, *p* = 0.07, η^2^_partial_ = 0.12, or Vision, *F*(1,19) = 1.94, *p* = 0.18, η^2^_partial_ = 0.08, but there was a significant interaction between these factors, *F*(2.02,38.36) = 5.28, *p* = 0.008, η^2^_partial_ = 0.22. Participants became less accurate in the normal vision condition across the 4 blocks, whereas they maintained a similar level of accuracy in the strobe vision condition. As shown in Fig. 2, constant error in the normal vision condition deteriorated from block 1 (−85 ms) to block 3 (-101 ms), *p* = 0.04, and block 4 (−103 ms), *p* < 0.01. As a consequence, while constant error was significantly less in the normal vision than strobe vision condition in blocks 1, there was no difference in constant error for the remaining blocks. Observation of the individual participants’ data revealed that 16 of 20 exhibited an increase in constant error between block 1 and 4 in the normal vision condition. In the strobe vision condition, an increase in error was evident in 9 of 20 participants.

**Figure 3:**
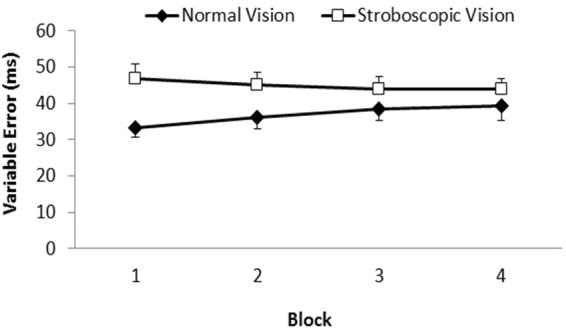
Group mean variable error as a function of time-on-task (Block 1–4) for the normal vision and stroboscopic vision conditions. Vertical bars represent standard errors of the mean.

For variable error there was a significant main effect of Acceleration, *F*(2,38) = 10.62, *p* < 0.001, η^2^_partial_ = 0.36. Participants were more variable when the object decelerated (45 ms) compared to when it moved with constant velocity (40 ms) or accelerated (38 ms), both *p* < 0.05. There was also a significant main effect of Vision, *F*(1,19) = 64.68, *p* < 0.001, η^2^_partial_ = 0.77, but this was superseded by a significant interaction between Vision and Block, *F*(3,57) = 3.04, *p* = 0.04, η^2^_partial_ = 0.14. As shown in Fig. 3, participants became more variable in the normal vision condition across the 4 blocks, whereas they maintained a similar level of variable error in the stroboscopic vision condition. As a consequence, the initial difference in variable error between the normal vision and stroboscopic vision conditions at block 1 (*p* < 0.01) and block 2 (*p < *0.03) was no longer present at block 3 and block 4 (*p* > 0.50). Observation of the individual participant data revealed that 15 of 20 exhibited an increase in variable error between block 1 and 4 in the normal vision condition. In the strobe vision condition, an increase in variable error was evident in 7 of 20 participants.

Additional analyses were conducted to determine if the change (between blocks 1 and 4) in mean and variability of coincidence-anticipation were related. This indicated no significant correlation in either normal vision, *r*(20) = 0.15, *p* = 0.54) or stroboscopic vision, *r*(20) = 0.40, *p* = 0.08). Notably, however, observation of the individual participant data revealed that 12 of the 20 became both less accurate and more variable in the normal vision condition. Only 1 participant became more accurate and less variable in the normal vision condition. The pattern was reversed in the stroboscopic vision condition, where 10 participants became more accurate and less variable. Of the remaining 10 participants, 6 became both less accurate and more variable.

### Psychomotor Vigilance Task

The analysis of the participants’ mean RT revealed a significant main effect of Block, *F*(2,38) = 6.99, *p* = 0.003, η^2^_partial_ = 0.27. Tukey HSD post-hoc tests indicated a significant difference between block 1 (268 ms) and block 3 (289 ms), *p* = 0.002. There was no main effect of Block on participants’ variability in RT, *F*(2,38) = 0.38, *p* > 0.68, η^2^_partial_ = 0.02. Group mean variability was 61, 53 and 67 ms, respectively. Additional analyses on the change in mean and variability of PVT indicated a significant relationship, *r*(20) = 0.59, *p* = 0.006. Observation of the individual participant data revealed that 9 of the 20 exhibited an increase in both mean and variability of RT. Only 3 participants reduced the mean and variability of RT between block 1 and block 3.

Finally, a generalised linear model with a poisson link function indicated the number of lapses was not influenced by Block (Wald Chi-Square = 1.37, *p* = 0.50), and thus did not provide a better fit of the data than the intercept-only model (Likelihood Ratio Chi-Square = 1.48, *p* = 0.48).

### Task Specificity

Pearson product-moment correlations were calculated to determine if there was a relationship between the change in performance (i.e., last block - first block) of coincidence-anticipation and PVT. There was no significant correlation between change in constant error and mean RT in the either normal, *r*(20) = *−*0.32, *p* = 0.18, or stroboscopic vision, *r*(20) = −0.03, *p* = 0.90. Still, observation of the individual participant data revealed that 13 of the 20 did in fact exhibit deterioration in constant error in the normal vision condition and an increase in RT. There was no significant correlation between change in variability of coincidence-anticipation and PVT in either normal, *r*(20) = 0.23, *p* = 0.34, or stroboscopic vision, *r*(20) = −0.90, *p* = 0.71.

## Discussion

It has recently been reported that practice under stroboscopic vision conditions can facilitate the development of sport-specific skill^2,21^, and that this could be explained in part by adaptation of processes such as motion coherence and attention in central vision^7^ and visual-spatial memory^5^. Central to this adaptation is the premise that practice in stroboscopic vision is effortful and demanding^20^. For instance, it has been reported that contrast sensitivity, which is important for form perception, is impaired at low levels of luminance^22^, and that thresholds for coherent motion (translational) and heading direction (radial) increase as luminance levels decrease^23^. Indeed, it is known that tracking a moving object relative to the surrounds in stroboscopic vision is an attentionally demanding task^9^, which likely engages areas in pre-frontal cortex associated with working memory for trajectory extrapolation^24^. In the current study, we adapted a method used to study psychomotor vigilance^12,20^, in order to determine if stroboscopic vision influences the ability to sustain attention to response accurately while performing a coincidence-anticipation task.

We found that the group of participants became less accurate and more variable in their coincidence-anticipation response in the normal vision condition as a function of time-on-task. Conversely, accuracy and variability were maintained a similar level in the stroboscopic vision condition. Consequently, differences in accuracy and variability that existed between normal vision and stroboscopic vision conditions during the first block of 30 trials were no longer evident during the last block of 30 trials. Consistent with explanations of the vigilance decrement, we interpret these data as showing that participants failed to sustain attention after repeated trials due to the monotony and relatively simple demands of coincidence-anticipation performed in normal vision (i.e., underload hypothesis^13^). At the level of individual participants, this was reflected in approximately two-thirds exhibiting deterioration in both accuracy and variability in the normal vision condition. Such a change in behaviour would not be expected if participants had developed a systematic bias (i.e., underestimation or overestimation) in their anticipation of object arrival time as a function of block.

In contrast, in the stroboscopic vision condition where there was a greater demand on visual-spatial memory for trajectory extrapolation, it would seem that participants were better able to sustain attention, and thus maintain performance over time. Indeed, there was evidence that some participants improved accuracy (n = 13) and variability (n = 11) across blocks in stroboscopic vision. For half of the participants there was a concurrent change in accuracy and variability that was consistent with a systematic improvement in anticipation of object arrival time. Importantly, this positive adaptation would not be expected had participants disengaged from the task due to high levels of boredom or fatigue. That is not to suggest, however, that coincidence-anticipation performance would be maintained indefinitely in the stroboscopic vision condition, and by all participants. Rather, in accord with the overload hypothesis^10^, it follows that a high attentional load and effortful processing cannot be maintained, thus eventually leading to the depletion of attentional resources and subsequently a vigilance decrement in performance.

An important consideration in previous work regarding the benefit of stroboscopic vision training has been the potential influence of motivational and expectancy effects such as placebo or Hawthorne^25^. In the current study, we were careful to include additional experimental control to ensure that any change in coincidence-anticipation was not simply a result of expectancy. In particular, given the use of novel eyewear for both the stroboscopic vision and normal vision conditions, there is no reason to believe that participants would have associated a particular eyewear with a treatment or control condition and thus modified their response accordingly. Also, we did not provide participants with knowledge of results, thus minimizing motivational effects of learning. This was important because there could have been asymmetrical motivational effects if participants were better able to use the knowledge of results in the normal vision condition to reduce response error to very low levels (e.g., 0–30 ms constant error previously reported^11^). Another important control was to present a real moving object rather than an apparent motion stimulus (e.g., Bassin-Anticipation timer). The idea was to minimize the possibility of asynchrony between the open state of the stroboscopic eyewear and presentation of the stimulus, thereby giving participants the opportunity to see the moving object for the duration of the 100 ms intermittent “open” interval. That said, it is worth noting that we were unable to equate the amount of light transmitted through the different eyewear, and thus reaching the eye, in the “open” state. Although a potential confound, we suggest that any difference in light transmission between the stroboscopic eyewear in the “open” state and the control eyewear is unlikely to have influenced the observed results. For instance, participants in our study reported being able to see normally through both eyewear (i.e., stroboscopic eyewear in the “open” state), whereas others have found that throwing and catching drills are not sufficiently demanding when the stroboscopic eyewear are set to level 1 (100 ms open, 67 ms closed)^5^. Finally, we also measured sustained attention in a computer-based vigilance task (PVT). This confirmed that the majority of participants became less accurate and more variable as a function of time-on-task. However, the vigilance decrement in the computer-based task was not significantly correlated with the change in coincidence-anticipation performance (i.e., accuracy and variability). This finding was not unexpected given that the two tasks have different processing and response demands, and consequently might be affected differently by the vigilance decrement^10,26^.

The notion that practicing coincidence-anticipation in stroboscopic vision engages attention is consistent with the “immediate benefit” reported by Smith & Mitroff^6^. In their study, participants’ coincidence-anticipation behaviour was significantly more accurate in a normal vision post-test immediately after practicing for 5–7 minutes in a stroboscopic vision condition compared to a normal vision condition. We concur with the authors’ suggestion that this effect was not evidence of long-term improvements due to learning, and instead that brief exposure to stroboscopic vision could be used to enhance performance when needed in specific game situations (i.e., before a baseball player prepares to bat). Interestingly, there is also some evidence that stroboscopic vision can be used to prevent and accelerate rehabilitation from injury^27^. However, as with studies that have shown improvements in psychomotor and function following stroboscopic vision training, it remains to be determined to what extent the benefits are due to an increase in attentional resource in order to cope with increased task difficulty and/or redirection of attention to alternative sources of information (e.g., somatosensory and vestibular inputs in the case of ACL injury).

Notably, while the current study used eyewear that are no longer available, there are alternative commercial eyewear (e.g., PLATO Visual Occlusion Spectacles; Senaptec Strobe; Visionup Strobe Glasses) that permit greater control over the duration of the open and closed states. While not the aim here, in future work it will be relevant to determine whether resistance to a vigilance decrement in stroboscopic vision is influenced by factors such as the amount of light transmitted through the lenses of the eyewear and the strobe rate, both of which could influence the perception of motion and form. For instance, we used a strobe robe of 4 Hz in the current study, but a lower strobe rate requiring longer intervals of extrapolation would potentially place greater demand on visual-spatial memory, thus more quickly leading to overload. Alternatively, practicing at a higher strobe rate requiring shorter intervals of extrapolation could quickly become less demanding, thus leading to disengagement. In this respect, the use of a “levelling-up” procedure whereby strobe rate is progressively reduced based on performance success^7^ would seem justified. Finally, an interesting question from the current study is whether alternating between periods of stroboscopic vision and normal vision during practice might have additional benefit. For instance, practice in stroboscopic vision might enable participants to offset the monotony of practicing in normal vision alone, thereby facilitating improved processing of relevant information and better learning.
